# Systemic Inflammation Impairs Attention and Cognitive Flexibility but Not Associative Learning in Aged Rats: Possible Implications for Delirium

**DOI:** 10.3389/fnagi.2014.00107

**Published:** 2014-06-10

**Authors:** Deborah J. Culley, Mary Snayd, Mark G. Baxter, Zhongcong Xie, In Ho Lee, James Rudolph, Sharon K. Inouye, Edward R. Marcantonio, Gregory Crosby

**Affiliations:** ^1^Department of Anesthesia, Harvard Medical School, Brigham and Women’s Hospital, Boston, MA, USA; ^2^Department of Neuroscience, Icahn School of Medicine at Mount Sinai, New York, NY, USA; ^3^Department of Anesthesia, Harvard Medical School, Massachusetts General Hospital, Boston, MA, USA; ^4^Department of Anesthesiology, Kwandong University College of Medicine, Cheil General Hospital, Seoul, South Korea; ^5^Department of Internal Medicine, Harvard Medical School, Brigham and Women’s Hospital, Boston, MA, USA; ^6^Department of Internal Medicine, Harvard Medical School, Beth Israel Deaconess Medical Center, Boston, MA, USA

**Keywords:** aging neuroscience, rats, inflammation, frontal cortex, lipopolysaccharides, CCL2, set-shifting

## Abstract

Delirium is a common and morbid condition in elderly hospitalized patients. Its pathophysiology is poorly understood but inflammation has been implicated based on a clinical association with systemic infection and surgery and preclinical data showing that systemic inflammation adversely affects hippocampus-dependent memory. However, clinical manifestations and imaging studies point to abnormalities not in the hippocampus but in cortical circuits. We therefore tested the hypothesis that systemic inflammation impairs prefrontal cortex function by assessing attention and executive function in aged animals. Aged (24-month-old) Fischer-344 rats received a single intraperitoneal injection of lipopolysaccharide (LPS; 50 μg/kg) or saline and were tested on the attentional set-shifting task (AST), an index of integrity of the prefrontal cortex, on days 1–3 post-injection. Plasma and frontal cortex concentrations of the cytokine TNFα and the chemokine CCL2 were measured by ELISA in separate groups of identically treated, age-matched rats. LPS selectively impaired reversal learning and attentional shifts without affecting discrimination learning in the AST, indicating a deficit in attention and cognitive flexibility but not learning globally. LPS increased plasma TNFα and CCL2 acutely but this resolved within 24–48 h. TNFα in the frontal cortex did not change whereas CCL2 increased nearly threefold 2 h after LPS but normalized by the time behavioral testing started 24 h later. Together, our data indicate that systemic inflammation selectively impairs attention and executive function in aged rodents and that the cognitive deficit is independent of concurrent changes in frontal cortical TNFα and CCL2. Because inattention is a prominent feature of clinical delirium, our data support a role for inflammation in the pathogenesis of this clinical syndrome and suggest this animal model could be useful for studying that relationship further.

## Introduction

Delirium, an acute neuropsychiatric syndrome, occurs commonly in hospitalized older medical and surgical patients. It is often precipitated by peripheral infection or inflammation induced by surgical trauma (Young et al., 1990; Wofford et al., 1996; Klausen et al., 1997; Broadhurst and Wilson, 2001; Buvanendran et al., 2006; Inouye, 2006; Nelson et al., 2006) and is important clinically because it is associated with poor functional outcome, a high rate of discharge to an extended care facility, a steeper trajectory of subsequent cognitive decline, and increased 1-year mortality (Greene et al., 2009; Smith et al., 2009; Saczynski et al., 2012). The pathogenesis of delirium is poorly understood, in part because there is no widely accepted animal model for examining mechanisms of the disorder. There are a multitude of putative causes of delirium but inflammation is a prime suspect based on clinical studies that reveal it is often precipitated in elderly patients by proinflammatory events such as infectious illness or surgery and laboratory evidence that cytokines and chemokines impair memory and leaning directly (Marcantonio et al., 2006; Rudolph et al., 2008; van Gool et al., 2010; Murray et al., 2012).

The difficulty, however, is that although there is little evidence for hippocampal dysfunction in delirium, most preclinical work on inflammation and cognition examines hippocampally mediated fear, reference, or working memory. Several cognitive domains can be affected in delirium but its pathognomonic feature is inattention. Efficient regulation of attention is thought to require close interaction between the prefrontal and parietal cortex, as well as neuromodulation from cholinergic basal forebrain neurons (Corbetta and Shulman, 2002; Sarter and Paolone, 2011). Hence, the inattention that occurs during delirium implies dysfunction of these cortical circuits. Recent functional imaging studies substantiate this view; in patients imaged during active delirium, the prefrontal cortex was hyper-active relative to the resting state. As such, behavioral tests mediated by the relevant cortical networks may be better suited than hippocampus-dependent behaviors to studying relationships between inflammation and a delirium-like phenotype in animals. The attentional set-shifting task (AST) is such a behavioral test. Developed for rats, the AST includes a set of discrimination problems based on stimulus dimensions such as odor and intra- and extra-dimensional cues and is analogous to the Wisconsin Card Sorting Test (WCST) in humans, which is used clinically to detect impairments in attention and executive function due to prefrontal cortex damage or dysfunction (Milner, 1963; Roberts et al., 1988). Here we used the AST to test the hypothesis that by promoting sustained neuroinflammation, systemic inflammation impairs attention and executive function in aged animals. We chose this task primarily because it can be rapidly acquired by aged rodents in contrast to other available tests of attention and executive function, for example, the 5-choice serial reaction time task, which require months of training and therefore pose a challenge for the study of aged animals.

## Materials and Methods

This protocol was approved by the Harvard Medical Area Standing Committee on Animals. A total of 31 24-month-old Fischer-344 rats were included; 11 were used for the behavioral part of the study and 20 were used for measurement of a representative cytokine and chemokine in plasma and brain. Systemic inflammation was induced by intraperitoneal (i.p.) administration of lipopolysaccharide (LPS; 50 μg/kg) while controls received an equal volume of saline i.p. LPS is a proinflammagenic protein from the cell wall of bacteria that is used widely to model systemic infection/inflammation. This dose was selected on the basis of published reports and preliminary studies showing that aged rats became ill but recovered within a few days.

Behavioral testing consisted of the AST. This procedure includes a sequence of discrimination problems in which rats dig for a food reward buried in one of two pots filled with digging medium (Birrell and Brown, 2000; Barense et al., 2002). The pots can be distinguished on several dimensions, including odor, type of digging medium, and texture applied to the outside of the pot. A sequence of problems is given in which only the relevant dimension is present (the simple discrimination, or SD), the irrelevant dimension is introduced alongside the same exemplars of the relevant dimension (the compound discrimination, or CD), new exemplars of all dimensions are introduced but the relevant dimension stays the same (the intradimensional shift, or IDS), and new exemplars of all dimensions are introduced but the relevant dimension changes (the extradimensional shift, or EDS). Problems are also reversed, such that the same stimuli are presented as the previous problem but the correct exemplar changes. An example sequence of problems is given in Table 1. In the present study, the two dimensions used were the digging medium filling the pot and a shape made of plastic foam placed on the wall of the maze adjacent to the pot. Thus demands on attention and executive function are introduced in several ways. Rats must adjust to changes in stimulus–reward contingencies in reversal problems, in which the previously correct stimulus is now incorrect, and vice versa, a condition that taxes behavioral flexibility. The EDS tests a different aspect of behavioral flexibility because the previously correct strategy for solving the discrimination problems (pay attention to digging medium, but not shape) must now be adjusted. These two kinds of behavioral shifts require the integrity of different regions of prefrontal and posterior parietal cortex (Dias et al., 1996; Birrell and Brown, 2000; McAlonan and Brown, 2003) and are sensitive to brain-wide blockade of muscarinic cholinergic receptors (Chen et al., 2004). As such, both the cognitive dimensions and brain region being assessed in the AST are relevant to human delirium.

**Table 1:** **Relevant dimension and positive discriminators used for each stage of the AST**.

	S+	S−	Relevant dimension
SD1	Paper vs.	Aspen	Medium
SD2	Heart vs.	Square	Shape
CD	Paper/flower vs. or paper/triangle	Aspen/triangle	Medium
		Aspen/flower	
CD-R	Aspen/flower vs. or Aspen/triangle	Paper/triangle	Medium
		Paper/flower	
IDS	Straw/diamond vs. or straw/cross	Cardboard/cross	Medium
		Cardboard/diamond	
IDS-R	Cardboard/diamond vs. or cardboard/cross	Straw/cross	Medium
		Straw/diamond	
EDS	Wax paper/star vs. or cotton/star	Cottar/circle	Shape
		Wax paper/circle	

Rats were acclimated to the behavioral testing procedure for 2–3 weeks prior to actual testing. Animals were food restricted to 85% of their baseline body weight during this time and were trained to dig by exposing them within the AST apparatus to pots containing rewards (half-Cheerios cereal), without discrimination clues. The AST apparatus was a large, clear plastic box (16 cm tall, 90 cm long, 44 cm wide) with a removable divider that separated the starting point for the rat from the pots. Testing procedures were similar to those published previously (Barense et al., 2002; Fox et al., 2003; Chen et al., 2004) except that the stimulus dimensions, rather than digging medium and odor, were digging medium and shape of a white plastic object placed on the wall immediately adjacent to the pot. Rats encountered the sequence of discrimination problems (Table 1) over four test sessions.

**Figure 1:**
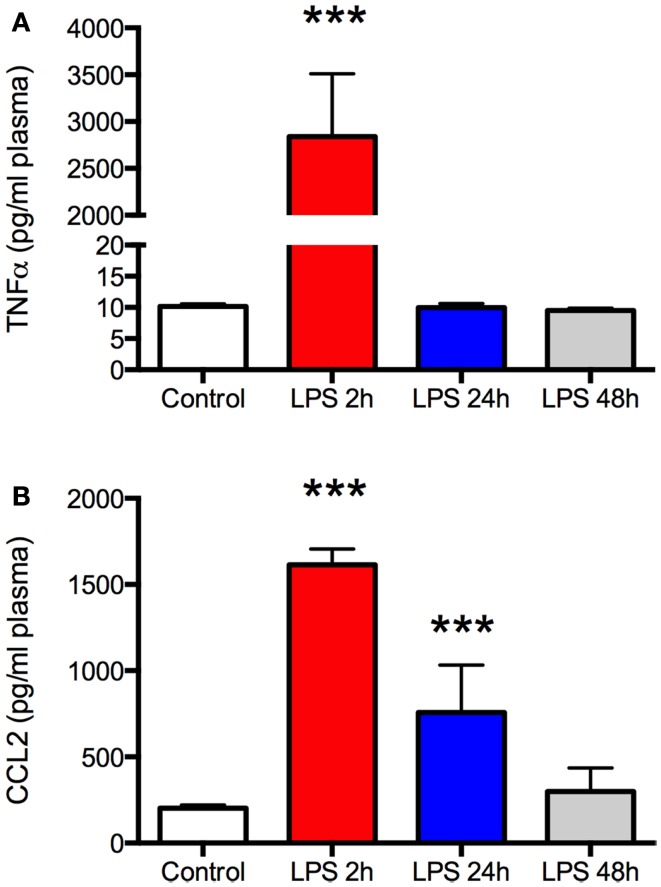
**Lipopolysaccharide (LPS) produces a robust but transient increase in plasma TNFα and CCL2**. Plasma was sampled at the time of sacrifice from aged Fisher 344 rats 2, 24, and 48 h after administration of 50 μg/kg LPS (*N* = 5 per group) or a control group (*N* = 5) that received an equal volume of saline intraperitoneally. There was a marked increase in both TNFα **(A)** and CCL2 **(B)** 2 h after LPS but the effect resolved completely by 24 or 48 h, respectively. Data are mean ± SEM. ***P < 0.001 by one-way ANOVA.

Once the animals were trained to dig reliably (defined as vigorous digging in six sequential trials in a row), the two SD tests were performed to establish a baseline (day 0; Figure 1). Then the rats were randomized to receive either LPS (*N* = 6) or saline (*N* = 5). The next day, each rat was tested on the CD and CD-R components of the AST; on day 2, rats were tested on the IDS and IDS-R components, and on day 3 they were each tested on the EDS. Each discrimination problem was presented until the rat reached performance criteria, defined as six consecutive correct trials. A “dig” was scored when the rat vigorously displaced the digging medium, because the reward was buried deeply within the pot. Thus, rats could investigate the digging medium with paws or snout before executing a “dig” response, and these choices were not scored. All media contained a small amount of powdered Cheerios reward, to mask the scent of the hidden reward. The order of relevant dimensions for the discrimination problems, and the assignment of specific stimuli to particular problems were the same between rats, to minimize intersubject variability. We recorded the number of trials to criterion for each discrimination problem (minimum of six) and this was the dependent variable for statistical analysis.

To assess the inflammatory response, we measured TNFα and CCL2 in the plasma and frontal cortex of a separate group of identically treated, age-matched rats (*N* = 5 LPS treated rats per group at 2, 24, or 48 h after injection and five controls) by commercially available ELISA kits (R&D Systems, Minneapolis, MN, USA). Both TNFα, a cytokine, and CCL2, a chemokine, are well-established mediators of inflammation and have been implicated in the pathogenesis of delirium (Rudolph et al., 2008; van Gool et al., 2010; Murray et al., 2012). Core blood was collected at the time of sacrifice and samples placed in EDTA-coated microcentrifuge tubes and centrifuged for 20 min at approximately 1000 × *g* within 30 min of collection. The plasma was then removed and stored at −20°C until used for measurement. Likewise, the brain was removed rapidly and frontal cortex frozen at −20°C in isopentane until assay. Assays were performed according to the manufacturer’s instructions, with the optical density of each sample determined using a microplate reader (SpectroMax2, Molecular Devices, Sunnyvale, CA, USA) at 450 nm with wavelength correction at 550.

Behavioral data were analyzed using a two-way ANOVA with treatment and trial number as the independent variables. ELISA data were analyzed with a one-way ANOVA comparing each time point to the control. Data are expressed as mean ± SEM and *P* < 0.05 was considered statistically significant.

## Results

**Figure 2:**
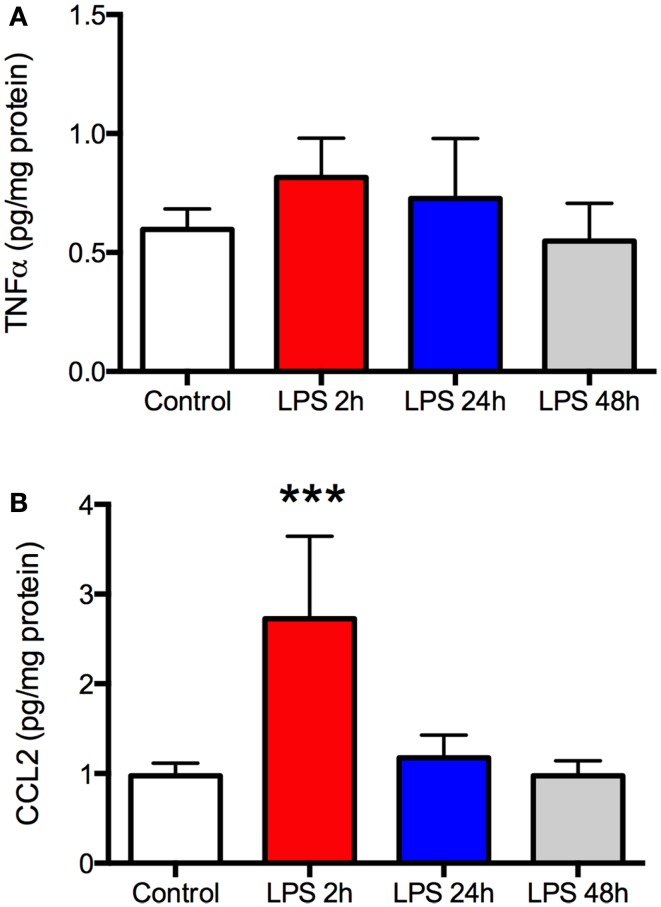
**Lipopolysaccharide (LPS) produces a robust but transient increase in TNFα and CCL2 in the frontal cortex**. Rats (*N* = 5 per group) were treated as described in Figure 1 and frontal cortex harvested at the time of sacrifice. There was no change in TNFα in the frontal cortex at any time compared to control animals **(A)**. CCL2 was elevated nearly threefold above control 2 h after LPS but this resolved by 24 h after treatment **(B)**. Data are mean ± SEM. ***P < 0.001 by one-way ANOVA.

As expected, there was a significant increase in plasma TNFα and CCL2 2 h after LPS (*P* < 0.001, Figures 1A,B), indicating a robust peripheral inflammatory response. This was transient, however, as plasma TNFα returned to baseline by the following day and CCL2 recovered within 48 h. Despite the changes in plasma, there was no increase in TNFα in the frontal cortex at any time (Figure 2A). In contrast, CCL2 was elevated nearly threefold in the frontal cortex 2 h after LPS but recovered to control levels within 24 h (Figure 2B). Thus, the peripheral and cortical inflammatory responses had resolved by the final day of behavioral testing.

**Figure 3:**
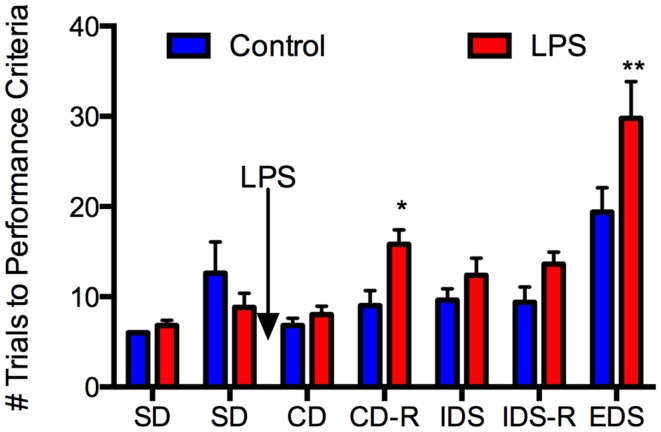
**Lipopolysaccharide selectively impaired attention/executive function in aged rats**. Twenty-four-month-old Fischer-344 rats (*N* = 11) were tested on two simple discrimination (SD) tasks, one from each dimension (medium and shape) prior to receiving LPS 50 μg/kg or an equal volume of saline i.p. They were tested on the compound discrimination (CD) task and compound discrimination reversal (CD-R) on day 1 after LPS, the intradimensional shift (IDS) and intradimensional shift reversal (IDS-R) on day 2 after LPS, and the extradimensional shift (EDS) on day 3 after LPS. There were no differences between the groups at baseline. LPS did not affect performance on the CD or IDS task but impaired performance on the CD-R and the EDS. This indicates LPS had no effect on simple discrimination learning but did impair attention/executive function for at least 3 days. Data are mean ± SEM. *P ≤ 0.05, **P ≤ 0.01 by two-way ANOVA.

With respect to behavior (Figure 3), repeated-measures ANOVA revealed a main effect of LPS treatment, *F*(1, 9) = 8.19, *P* = 0.019, a main effect of test phase, *F*(6, 54) = 22.56, *P* < 0.0005, and an interaction of these two factors, *F*(6, 54) = 3.12, *P* = 0.011. Simple main effects analysis revealed significant effects of LPS treatment on the CD-R, *F*(1, ~62) = 6.35, *P* = 0.014, and the EDS, *F*(1, ~62) = 15.34, *P* = 0.0002, and a trend toward an effect on the IDS-R, *F*(1, ~62) = 3.66, *P* = 0.06, but no significant effects or trends on any other phases, *F*s(1, ~62) < 1.77, *P* > 0.188. Thus, LPS treatment selectively impaired discriminations that required a shift of attention between sensory dimensions of the problem (the EDS) or a reversal of stimulus–reward contingencies (the reversals), but was without effect on SD learning, even when an irrelevant stimulus dimension was present.

In the control rats, the EDS discrimination was significantly more difficult than the IDS, *t*(4) = 5.82, *P* = 0.004 (Figure 3), indicating that an attentional set was formed, because although both discriminations were between novel stimuli, only the EDS required a shift of attention to a different stimulus dimension. Notably, neither the CD-R nor the IDS-R were significantly more difficult than the IDS in control rats, each taking about nine trials to learn to criterion, *t*s(4) < 0.22, *P* > 0.84. Thus, the effect of LPS on the reversal problems (at least) is not simply because these problems are more difficult, as the equally difficult IDS was unaffected by LPS treatment.

Because all the rats experienced a shift in the same direction, from digging medium to shape, it could be argued that poorer performance on the EDS reflects a more difficult discrimination problem rather than a shift in attention. Note, however, that all rats had solved SDs with each dimension in the first phase of training. Although SD2 (shape) is more difficult than SD1 (medium) when performance is analyzed in the entire group of rats [SD1 mean 6.36, SD mean 10.73, *t*(10) = 2.52, *P* = 0.031], the EDS is more difficult than SD2 in both control [*t*(4) = 4.65, *P* = 0.010] and LPS [*t*(5) = 5.75, *P* = 0.002] groups. Thus, performance in the EDS reflects a cost of shifting attention between medium and shape, even though the two dimensions are not necessarily matched for difficulty.

## Discussion

To our knowledge, this is the first study to document that systemic inflammation impairs attention and executive function specifically. This impairment was selective to the extent that associative learning was not affected, as aged rats challenged with LPS were able to learn discrimination problems presented in the AST as well as those treated only with saline. However, when the task became more complex and demanded more cognitive flexibility and greater attention/executive function – reversing stimulus–reward associations or shifting the focus of attention to a different perceptual quality of the stimuli – the aged rats challenged with LPS were impaired. This is consistent with a phenotype of cognitive inflexibility and impaired attention/executive function and shows the behavioral disability cannot be explained by lack of motivation, malaise due to “sickness behavior,” or a global, non-specific impairment in CNS function. Moreover, while LPS induced a robust inflammatory response, the attentional deficit was present even after the systemic and frontal cortical proinflammatory changes resolved. As such, it appears that systemic inflammation triggers impairment of fronto-cortically-mediated aspects of cognition in aged rats but is not required to sustain it. To the extent that inattention is a defining characteristic of delirium, our results support a role for inflammation in the pathophysiology of this condition and suggest attentional set-shifting is a promising behavioral paradigm for investigating this relationship more closely (Inouye, 2006; Greene et al., 2009).

The AST challenges attention and executive function in several ways. Rats must adjust to changes in stimulus–reward contingencies in reversal problems, in which the previously correct stimulus is now incorrect, and vice versa, thus taxing behavioral flexibility. A different aspect of behavioral flexibility is tapped in the EDS, because the previously correct strategy for solving the discrimination problems (pay attention to digging medium, but not shape) must now be adjusted. These two kinds of behavioral shifts require the integrity of different regions of prefrontal cortex (Dias et al., 1996; Birrell and Brown, 2000; McAlonan and Brown, 2003). Moreover, both are sensitive to brain-wide blockade of muscarinic cholinergic receptors (Chen et al., 2004), a strategy that has also been used to model delirium (Trzepacz et al., 1992). As such, much like the WCST in humans, this task allows testing of the impact of an insult such as systemic inflammation or surgery on attention and executive function, as distinct from learning *per se*. In fact, our data show that deficits in attention and learning are dissociable during systemic inflammation. Consequently, the AST assesses a cognitive phenotype analogous to that of acute illness-related cognitive morbidity in humans.

Numerous clinical studies show an association between infectious illness, surgery, and elevated plasma proinflammatory cytokine and chemokine concentrations and delirium (Inouye, 2006; Rudolph et al., 2008; van Gool et al., 2010; Rudolph and Marcantonio, 2011; Murray et al., 2012). Likewise, preclinical studies establish that inflammation produces cognitive deficits. For example, others have reported that systemic administration of LPS produces a robust immune response, deficits in learning, and “sickness behavior” similar to that observed in ill humans (Barrientos et al., 2006; Neri et al., 2006; Sparkman et al., 2006; Murray et al., 2012). Proinflammatory mediators such as TNFα and CCL2, which were upregulated in the plasma and frontal cortex here, are potent inhibitors of long-term potentiation (an *in vitro* model of memory) as well as learning in hippocampally mediated cognitive tests in intact animals and are elevated in the brain of patients suffering from dementia (Tancredi et al., 2000; Vereker et al., 2000; Barrientos et al., 2006; Neri et al., 2006; Sparkman et al., 2006; Holmes et al., 2009; Nelson et al., 2011; Westin et al., 2012). The problem, however, is that delirium appears to reflect dysfunction not in the hippocampus but in the prefrontal cortex and cortical–subcortical networks.

Delirium is a cognitively complex syndrome but inattention is its hallmark feature (Inouye, 2006; Rudolph and Marcantonio, 2011). This points to abnormalities in the prefrontal cortex because this region is essential for normal attention (Corbetta and Shulman, 2002; Sarter and Paolone, 2011). Moreover, recent imaging studies of the active phase of delirium reveal abnormal functional connectivity between the dorsolateral prefrontal cortex and the posterior cingulate cortex, as well as between cortical and subcortical regions, but no abnormalities in functional connectivity of the hippocampus (Choi et al., 2012). Further, most animal models of delirium, including those investigating the role of inflammation, have employed hippocampus-dependent behavioral tasks. For example, contextual fear conditioning (CFC) experiments show clearly that infection and surgery-induced inflammation impair performance but the applicability of this paradigm to delirium is probably limited because CFC assesses function of the hippocampus and amygdala (Kennard and Woodruff-Pak, 2011) and impairment is detected even in healthy young animals under conditions of mild inflammation (Cibelli et al., 2010; Terrando et al., 2010; Cunningham and Maclullich, 2013). Inflammation also disrupts reference and working memory under some conditions. Thus, animals challenged with doses of LPS similar to that used here perform normally on a *Y* - or *T*-maze alternation task a few hours later but perform poorly on a working memory matching-to-place version of the Morris water maze, which requires animals to integrate new information with existing memories (Chen et al., 2008; Murray et al., 2012). As these are hippocampally mediated tasks, it is evident that inflammation produces acute hippocampal dysfunction and cognitive inflexibility. These considerations motivated our use of a test of attention and executive function in this study that relies on the prefrontal cortex rather than the hippocampus.

To the extent that the ability to solve discrimination problems that required a shift of attention between sensory dimensions or stimulus–reward contingencies (the reversals) was impaired but associative learning was intact, our data show that in aged rodents systemic inflammation selectively impairs attention and, by inference, fronto-parietal circuits without affecting hippocampal networks. This differential effect is interesting in light of recent evidence that crystallized cognition is preserved in human delirium whereas fluid processing is impaired (Brown et al., 2011). Third, in contrast to the concurrent normalization of the inflammatory and behavioral responses observed in most models using hippocampal-mediated tasks, the behavioral deficit on AST testing persisted after the inflammatory response resolved, suggesting that frontal–cortical and hippocampal circuits are differentially sensitive to neuroinflammation. Together, these data add strong support for the hypothesis that systemic inflammation causes a delirium-like phenotype in aged rodents.

Nonetheless, this study is limited in a few important respects. Impaired performance in the AST could be the result of inattention (moment to moment deficiencies in orienting to discrimination problems) and/or impaired executive function (higher-order deficits in changing behavioral strategies) so our results could reflect defects in both cognitive domains. The task does, however, test the prefrontal cortex, which is a region implicated in the pathogenesis of delirium, and does not involve the hippocampus (Brooks et al., 2012). Delirium can occur in hospitalized young adults but it is rare; therefore, we did not include young adult rats in this study. Extrapolation of results in rodents to a behaviorally complex and heterogeneous clinical condition such as delirium, a pathophysiologically poorly characterized syndrome, must also be made cautiously. Delirium affects cognitive domains beyond attention and executive function, including memory, arousal, and affect, so a deficit in attention or executive function yields only an incomplete picture of the clinical syndrome. In addition, we examined just two representative proinflammatory mediators of the several that have been implicated in delirium pathogenesis (Rudolph et al., 2008; van Gool et al., 2010; Murray et al., 2012; Cunningham and Maclullich, 2013). As such, although TNFα and CCL2 returned to normal levels by the time of behavioral testing, we cannot exclude a role for ongoing neuroinflammation in the behavioral deficits we observed because abnormalities could be signaled by other mediators of inflammation. Finally, we did not assess cognitive recovery and therefore cannot exclude the possibility that the behavioral features we observed reflect the early stages of an inflammation-induced chronic neurodegenerative process, as opposed to a transient or fluctuating disruption of neural circuitry and function by inflammatory mediators.

In conclusion, our data show that LPS-induced systemic inflammation selectively impairs the ability of aged rodents to attend to and process environmentally salient stimuli and does so without global cognitive dysfunction or a persistent increase in TNFα or CCL2 expression in the frontal cortex. Inasmuch as inattention is a key diagnostic feature of clinical delirium, our data support a role for inflammation in the pathogenesis of this clinical syndrome and suggest this animal model could be useful for studying that relationship further.

## Conflict of Interest Statement

The authors declare that the research was conducted in the absence of any commercial or financial relationships that could be construed as a potential conflict of interest.
